# Inductive Tracking Methodology for Wireless Sensors in Photoreactors

**DOI:** 10.3390/s21124201

**Published:** 2021-06-18

**Authors:** David Demetz, Alexander Sutor

**Affiliations:** Institute of Measurement and Sensor Technology, UMIT-Private University for Health Sciences, Medical Informatics and Technology, Eduard-Wallnöfer-Zentrum 1, 6060 Hall in Tirol, Austria; alexander.sutor@umit.at

**Keywords:** inductive localization, inductive data exchange, wireless sensors, photoreactor

## Abstract

In this paper, we present a methodology for locating wireless sensors for the use in photoreactors. Photoreactors are, e.g., used to cultivate photosynthetic active microorganisms. For measuring important parameters like, e.g., the temperature inside the reactor, sensors are needed. Wireless locatable floating sensors would enable it to measure the data anywhere inside the reactor and to get a spatial resolution of the registered data. Due to the well defined propagation properties of magnetic fields and the fact that they are not significantly influenced in underwater environments when using low frequencies, a magnetic induction (MI) system is chosen for the data transmission as well as for the localization task. We designed an inductive transmitter and a receiver capable of measuring the magnetic field in every three spatial directions. The transmitting frequency is set at approx. 300kHz. This results in a wavelength of approx. 1km which clearly exceeds the dimensions of our measurement setup where the transmitter–receiver distances in general are lower than one meter. Due to this fact, only the quasi-static field component has to be considered and the location of the transmitter is calculated by measuring its magnetic field at defined positions and in using the magnetic dipole field equation in order to model its magnetic field geometry. The used measurement setup consists of a transmitter and two receivers. The first measurements were performed without a water filled photoreactor since no differences in the propagation criteria of magnetic fields are expected due to the negligibly low differences in the relative magnetic permeability of water and air. The system is calibrated and validated by using a LIDAR depth camera that is also used to locate the transmitter. The transmitter positions measured with the camera are therefore compared with the inductively measured ones.

## 1. Introduction

### 1.1. Context and Motivation

**Figure 1:**
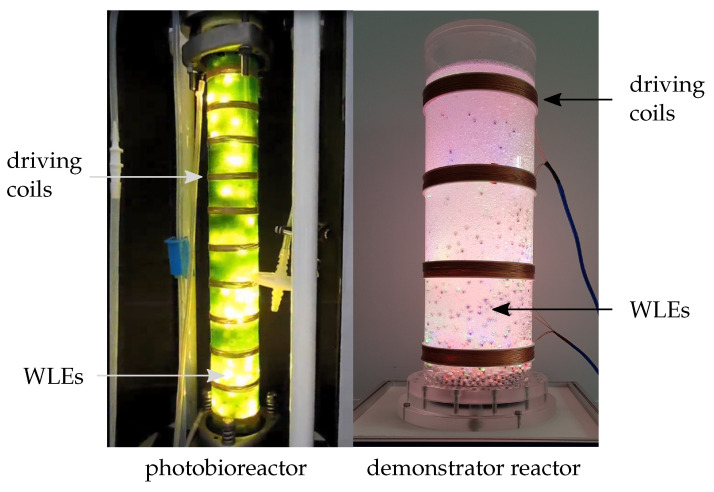
Internal illumination of reactors by means of the WLE based internal illumination system.

Photoreactors are widely used, e.g., for the cultivation of phototrophic microorganisms such as cyanobacteria, whose pigments phycoerythrin and phycocyanin gain in importance in the cosmetic, food, or pharmacy industries [1,2]. The development of an effective scale-up of the photobioreactors in order to also achieve the same functionality of the laboratory scale reactors for the large scale photobioreactors is a challenge [3]. The main hurdle is the limited penetration depth of light, the dominant parameter affecting the grow rate of microalgae cultures [1]. In order to increase the ratio between the illuminated surface and the reactor volume, the used reactors are characterized by small layer thicknesses, which lead to a significant increase in the construction volume [4]. To counteract this issue, a wireless internal illumination for photoreactors has been developed [1]. The system mainly consists of small glowing spheres called wireless light emitters (WLEs) that are energized wirelessly from outside the reactor through an inductive link. To allow the WLEs to float in the reactor media, the overall density of one single WLE is in the range of water. The WLEs are distributed in the reactor due to the air lift of the reactor. The driving coils used to produce the magnetic field are mounted at the outer diameter of the photoreactor and are driven by a class-E amplifier. The generated magnetic flux density is approx. 1mT at a frequency of 178kHz [1,4,5]. The internal illumination system based on the WLEs is also used to perform photocatalytic reactions; therefore, the WLEs are coated by a photocatalyst [6]. Another area of application for the internal illumination system is the photobiosynthetic production of biodiesel from fatty acids where the WLEs are used to intensify the process [7]. Figure 1 shows the WLE based internal illumination system in a photobioreactor and in a demonstrator reactor.

More insight into the situation inside the reactors is actually missing, and increased measurement and control could also be helpful in already existing plants [8]. For measuring parameters inside the reactor, they have to be equipped with sensors. Commonly monitored parameters in micro algal photobioreactors are the temperature, light intensity, the pH value, and the dissolved oxygen [9].

### 1.2. State-of-the-Art Setups and the Expected Benefits of Using Wireless Sensors

In a state-of-the-art setup, those sensors are mounted at fixed positions inside the reactor [9]. Drill holes and cable guides are therefore required and the measured values are only available at one position in the reactor. The use of floating wireless sensors would make it possible to register the data anywhere inside the reactor. In addition, the reactors would not have to be structurally changed, and the measurement system would also be easily scalable. A suitable energy supply for the sensors has to be chosen. Kanoun et al. [10] reviewed methods for an efficient energy supply of wireless sensor nodes. The use of a wireless power transfer provides benefits as e.g., a battery-free sensor node design. Additionally, it shows great potential for non-accessible systems [10]. Due to the aforementioned advantages and the already existing inductive power supply for the internal illumination, which can also be used for the sensors, our sensors are also planned to be supplied wirelessly with energy. The data transmission will also be realized through an inductive link because of the promising propagation properties of magnetic fields in an underwater environment.

### 1.3. Structure of This Paper

This paper is structured as follows. Section 2 discusses the data transmission in underwater environment by taking a closer look at several physical properties of the three main data transmission methods, especially the electromagnetic data transmission. The analytical equations used to localize the transmitter by measuring its magnetic field at defined positions are described in Section 3. Those equations are validated by a finite element simulation where the magnetic field of our transmitter coil is simulated. The comparison between the simulation results and the analytical model is also shown in Section 3. The developed hardware is described in Section 4, where the used transmitter design, the receiver design, and the laboratory setup used to perform location measurements are presented. The calibration method used to calibrate the receivers is shown in Section 5. Furthermore, the measurement results are displayed in Section 6 and discussed in Section 7 where also some possible improvement suggestions are presented. The conclusions are given in Section 8.

## 2. Data Transmission in an Underwater Environment

In order to wirelessly transmit the sensed data from inside the water/saltwater filled reactor, a suitable data transmitting method has to be chosen. According to Che et al. [11], there are three main categories for the underwater wireless data exchange:

- Optical data transmission;
- Acoustic data transmission;
- Electromagnetic data transmission.

### 2.1. Acoustic and Optical Data Transmission in Underwater Environments

The acoustic data transmission is a proven technology for long ranges up to 20km. However, the technology suffers from a low bandwidth and signal reflections at boundaries. The acoustic method is therefore not well suited for our operation environment because of the physical separation due to the reactor wall between the sensor (transmitter) inside the reactor and the receiver placed outside. This would cause the acoustic signals to be reflected. The optical data transmission achieves high bandwidths, but, because of the susceptibility to turbidity and particles, this method is also unsuitable for our use [11,12].

### 2.2. Physical Properties of the Radio Frequency Electromagnetic Communication in Underwater Environments

Compared to air, the propagation properties of electromagnetic waves differ significantly in water. This because of its high permittivity and the electrical conductivity [11]. The electric field component, which is assumed to be the *x*-component of the electromagnetic wave with the amplitude $E_{0}$ propagating in the positive *z*-direction through a conducting media, is described by the following equation ([13] p. 138):

(1) $$E_{x}\left(z\right)=E_{0}e^{-\alpha z}e^{-j\beta z},$$

where α is the attenuation constant and β the phase constant. The attenuation constant α is described by Equation (2) ([13] p. 139):

(2) $$\alpha =\omega \sqrt{\mu \varepsilon }{\left[\frac{1}{2}\left(\sqrt{1+{\left(\frac{\sigma }{\omega \varepsilon }\right)}^{2}-1}\right)\right]}^{\frac{1}{2}}\;Np/m.$$

The conductivity of the propagation medium is referred to as σ, the permittivity as ε, the permeability as μ, and the angular frequency as ω. Equation (2) can be simplified for two main electrical properties of the propagation medium ([13] p. 142):

- good dielectric: ${\left(\frac{\sigma }{\omega \varepsilon }\right)}^{2}\ll 1$
  (3) $$\alpha \approx \frac{\sigma }{2}\sqrt{\frac{\mu }{\varepsilon }},$$
- good conductor: ${\left(\frac{\sigma }{\omega \varepsilon }\right)}^{2}\gg 1$
  (4) $$\alpha \approx \sqrt{\frac{\omega \mu \sigma }{2}}.$$

The conductivity of the propagation medium has to be investigated in order to choose the right approximation equation for the attenuation constant α. Seawater with an average conductivity value of around 4S/m is considered as a high-loss medium [12,14]. The attenuation constant is therefore calculated with Equation (4), and the value of α increases with the higher frequency and conductivity.

The conductivity of freshwater on the other hand is about 400 times lower compared to seawater [12], and its attenuation constant can therefore be calculated with Equation (3). It can be seen that, in this case, the approximated attenuation coefficient is independent of the frequency but is directly subjected to changes in the conductivity.

### 2.3. Magnetic Induction (MI) Data Transmission

Since the radio frequency electromagnetic data transmission suffers from high attenuation in the underwater environment, we decided to take a closer look at the near field magnetic induction approach for the data transmission using low frequencies. The transmitter can be modeled as a quasi static magnetic field source if the wavelengths are much longer than the photoreactor dimensions [15].

The magnetic flux density B is generally calculated with the following Equation:

(5) $$B=\mu_{0}\mu_{r}H,$$

where $\mu_{0}=1.25663706212\left(19\right)\times 10^{-6}\;N/A^{2}$ is the magnetic permeability, $\mu_{r}$ the material dependent relative permeability, and H the magnetic field strength of the source. Due to the negligibly low difference between the relative permeability $\mu_{r}$ of water and air ($\mu_{r-water}=0.999991$; $\mu_{r-air}=1.0000004$ ([13] p. 51)), the propagation properties of quasi-static magnetic fields do not differ between these media. Because of this, the magnetic induction (MI) looks promising for the use in our application. Using low frequencies, the wavelengths greatly exceed the dimensions of our setup (e.g., at a frequency of 300kHz the wavelength is 1km) and therefore only the quasi static field components have to be taken into account [15]. Hence, the attenuation factor α does not matter in this case.

In general, loosely coupled MI systems for data and power transmission are widely used in medical implants like cochlear implants, pacemakers, or retinal implants [16]. In [17], they use the MI system as a communication link between two hearing devices since the relative magnetic permeability of human tissue does not differ significantly from the relative magnetic permeability of water or air.

**Figure 2:**
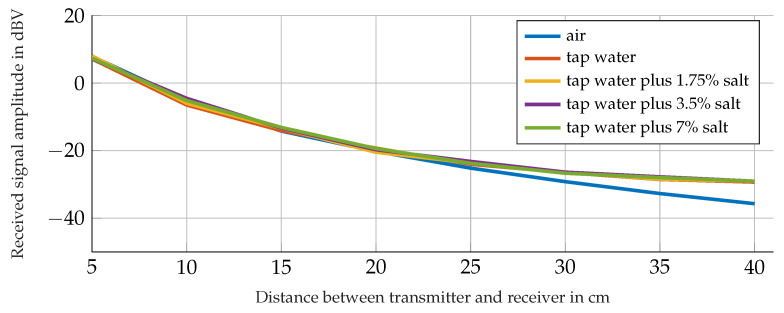
Receiver signal amplitudes for different propagation media and receiver–transmitter distances [18].

In order to prove that there are practically no differences in the transmitting performance for tap water, salty water in different salinities and air as propagation media, we performed some test measurements with a transmitter coil placed inside a plastic box filled with the named media and and a receiver coil mounted outside, both aligned to the same axis. Figure 2 shows the measured amplitudes at the receiver side for different distances between the transmitter and receiver and for the named propagation media. The transmitter coil is therefore driven with a sine signal at a frequency of 295kHz. The receiver is an LC-tank tuned to the transmitting frequency with the receiver coil connected in parallel to a capacitor and a resistance [18]. Figure 2 shows the results of those measurements.

We choose the MI method as a data transmission system for our aim since it shows no differences in performance between the listed propagation media.

## 3. Localization Method

The propagation properties of the magnetic field of a coil have to be investigated carefully in order to enable the determination of its position by measuring its magnetic field at defined positions. In this section, an analytical method to describe the magnetic field of a coil is compared to simulation results. We use the software *COMSOL Multiphysics* (5.4, Stockholm, Sweden) with the *AC/DC Module* to simulate the magnetic field of our transmitter coil using its real dimensions and properties. The simulation results are compared to the analytical ones in order to validate the analytical model used for our purposes. The validated analytical equations are used in Section 3.3 to derive the localization methodology.

### 3.1. Analytical Description of the Magnetic Field of the Transmitting Coil

**Figure 3:**
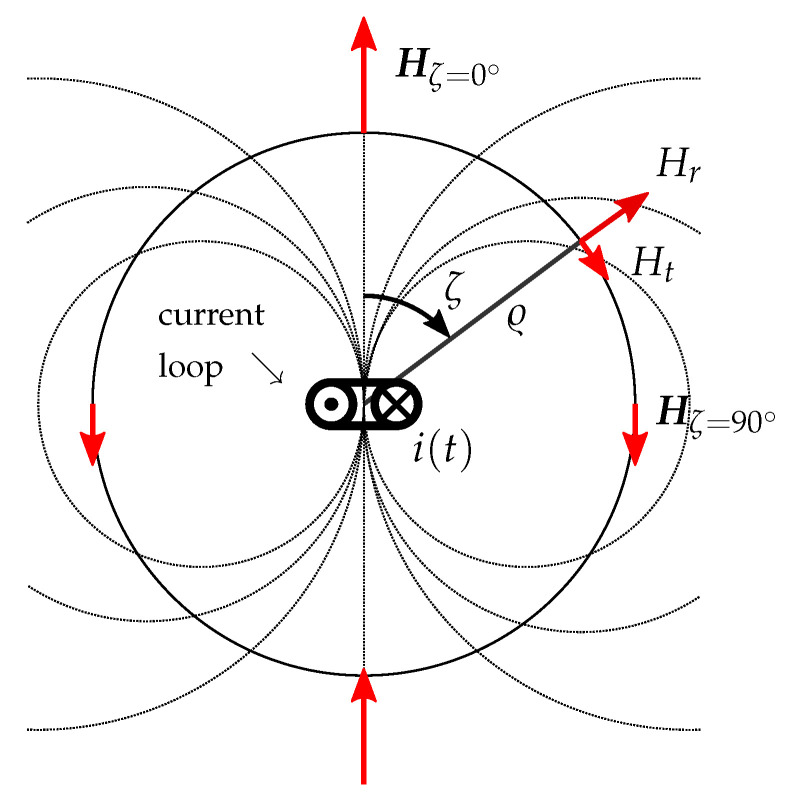
Magnetic dipole field [15].

If the transmitter coil dimensions are small compared to the distance between the coil and the point where its magnetic field is measured, it can be modeled as a magnetic dipole point source. Like mentioned in Section 2.3, only the quasi static component of the magnetic field has to be considered if the wavelength is long compared to the distance ϱ between the transmitter and the measuring point (ϱ≪λ/2π) [15], as it is the case with our setup. The model equations of the transmitter coil magnetic field are therefore defined as follows [15]:

(6) $$H_{r}=\frac{NIA}{2\pi \varrho^{3}}\cos\zeta ,$$

(7) $$H_{t}=\frac{NIA}{4\pi \varrho^{3}}\sin\zeta ,$$

where $H_{r}$ describes the radial component and $H_{t}$ the tangential component of the magnetic dipole field at the distance ϱ from the coil center (see, therefore, Figure 3). ζ is the angle between the coil axis and the point in which the magnetic field is calculated. The number of turns of the transmitting coil is referred to as *N*, the cross-section area of the coil as *A* and the exciting current amplitude as *I* with i(t)=Icosωt.

### 3.2. Simulation of the Magnetic Field and the Comparison between the Simulation Results and the Analytical Calculation

We performed finite element simulations with *COMSOL Multiphysics* to validate the analytical approximation used to calculate the magnetic field of our transmitting coil. The cylindrical transmitter coil with a radius of 1cm and a length of 2cm and 115 turns is therefore modeled in *COMSOL Multiphysics* as a 3D model. In order to simulate its magnetic field, the *Magnetic Fields (mf)* physics interface is used. The electrical material parameters for the coil body, which in common used coils is made of plastic, are set to the following values (for polyethylene): the conductivity was set to $10^{-15}\;S/m$ [19], the relative permittivity to 2.26 [13], and the relative permeability to 1 [20]. The surrounding coil volume is given the material properties of fresh water with a relative permeability of 0.999991, a relative permittivity of 81, and a conductivity of 0.01S/m. These values were taken from [13]. The coil is simulated as a *Homogenized multi-turn Coil* according to the *Magnetic Fields (mf)* physics interface of *COMSOL Multiphysics*. Therefore, the coil wire conductivity is set to $5.76\times 10^{7}\;S/m$ (copper) [13] and the coil wire cross section area to $4\times 10^{-8}\;{\mathrm{mm}}^{2}$ (which corresponds to the wire used in our real transmitter coil). The used mesh is a tetrahedral mesh. We evaluate the magnetic field of the simulated coil for different radial distances around the coil center on a plane defined by the coil axis and the coil radius. Additionally, the magnetic field is also calculated for those radial distances in using the analytical approximation described with Equations (6) and (7). Both results are unity-based normalized in order to get amplitudes between 0 and 1. This enables a comparison between the simulation results and the calculated values. A comparison using normalized values is justified since we calculate the position of our transmitter in evaluating the geometry of the magnetic field and not using absolute amplitude values (see, therefore, the next Section 3.3). The Normalized Root-Mean-Square Error (NRMSE) was calculated between the magnetic field values calculated using the magnetic dipole equation and the simulated ones. Therefore, the software *MATLAB* (2020b, MathWorks, Natick, MA, USA) was used. According to its documentation, the NRMSE value is calculated with the following equation:

(8) $$\mathrm{NRMSE}=\sqrt{\frac{\sum_{{}_{i}}{\left(x_{r_{i}}-x_{{}_{i}}\right)}^{2}}{\sum_{{}_{i}}{\left(x_{r_{i}}-\bar{x_{r}}\right)}^{2}}}.$$

In our case, the reference data array $x_{r}$ would be the simulated magnetic field and the data array *x*, which is compared to the reference, would be the calculated magnetic dipole field ($\bar{x_{r}}$ is the mean value of the reference data).

**Figure 4:**
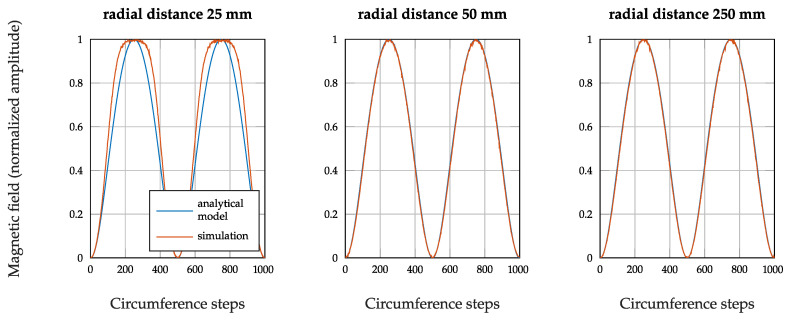
Comparison between the simulated magnetic field shape of the transmitter coil and the analytically calculated one.

A mesh independence study was performed in order to guarantee the maximal independence of the simulation results from the discretization. Therefore, the physics controlled mesh size was stepwise reduced, from *fine* to *extremely fine* and further on by reducing manually the maximal and minimal element size until no significant changes in the NRMSE result were noticed. The values presented here are taken from the simulation performed with the finest mesh. Figure 4 shows the comparison of the simulated and calculated magnetic fields for different radial distances from the coil center.

**Figure 5:**
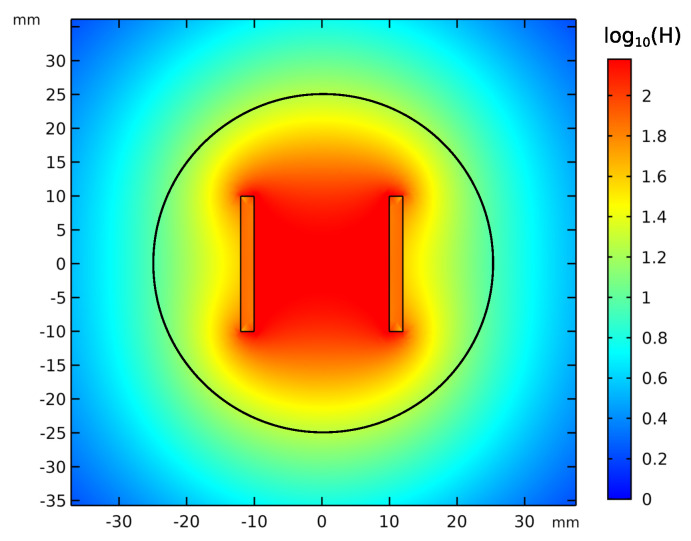
Simulation in *COMSOL*: magnetic field magnitude in $\left(log_{10}\frac{H}{1\frac{A}{m}}\right)$.

Figure 5 shows the magnitude of the magnetic field evaluated on the plane described, and the black circle in Figure 5 is the curve for which the simulated magnetic field is compared to the calculated one in Figure 4 for a radius of 25mm. Since the magnetic field decreases with the power of three of the distance from the coil center, the colorbar in Figure 5 is scaled with the logarithm of ten in order to better see the field course.

Table 1 shows the calculated NRMSE values for the three different radial distances.

The deviations between the simulation and the analytical model decrease with increasing radial distance. The radial distance of 250mm best reflects the order of magnitude of the real distances in our measurement setup. For this distance, the NRMSE value is 0.0258, which corresponds to a fit percentage of 97.42%. This validates the analytical formula used to describe the magnetic field of the transmitting coil.

### 3.3. Coupling Equation between the Transmitter Coil and a Receiver Which Is Able to Measure the Magnetic Field in All Three x-, y-, and z-Directions

**Figure 6:**
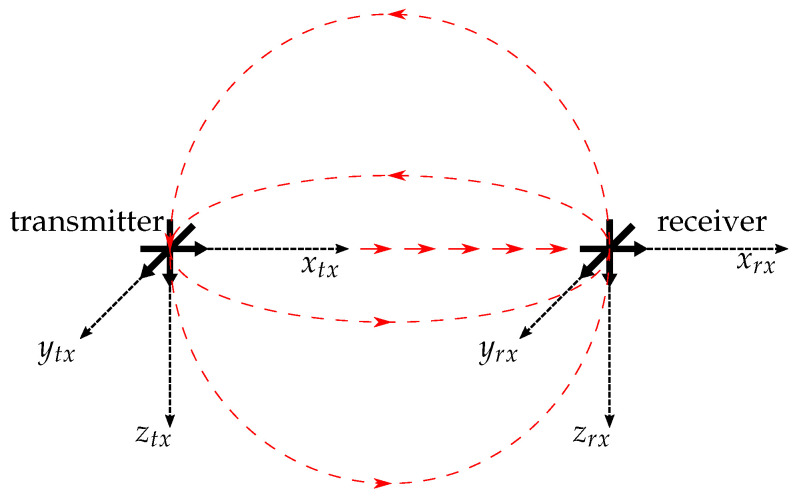
Aligned transmitter and receiver [15].

Since the differences between the results of the analytical calculations by using Equations (6) and (7) and the results of the finite elements simulations in Section 3.2 are negligibly low, Equations (6) and (7) are used to define the analytical relationship between the transmitter and a receiver. According to Raab et al. [15], the equation describing the inductive coupling of a triaxial transmitter with an aligned triaxial receiver like that shown in Figure 6 can be described as follows:

(9) $${\vec{f}}_{rx}=\left(\frac{C}{\varrho^{3}}\right)S{\vec{f}}_{tx},$$

with

S=diag(1−0.5−0.5).

*C* is a constant factor which depends from receiver parameters like the gain factor and the coil properties and ϱ the distance between the transmitter and the receiver. ${\vec{f}}_{tx}$ is the transmitter signal vector and ${\vec{f}}_{rx}$ the receiver signal vector. The matrix *S* in Equation (9) is derived from Equations (6) and (7) for the following alignment conditions, which are also shown in Figure 6:

$$\begin{array}{c} {\vec{e}}_{x-rx}={\vec{e}}_{x-tx},\\ {\vec{e}}_{y-rx}\|{\vec{e}}_{y-tx},\\ {\vec{e}}_{z-rx}\|{\vec{e}}_{z-tx}. \end{array}$$

The *x*-axes of the transmitter and the receiver are only coupled via the radial component of the magnetic field (see, therefore, Equations (6) and (7) and Figure 3 at an angle of $\zeta =0^{\circ }$) and the *y*- and *z*-axes only via the tangential component (see therefore Equations (6) and (7) and Figure 3 at an angle of $\zeta =90^{\circ }$).

**Figure 7:**
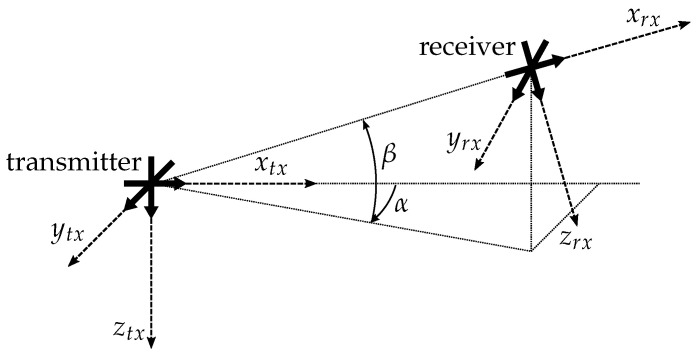
Transmitter–receiver arrangement expanded by the angles α and β [15].

In reality, the transmitter and the receiver are not aligned like that described in Figure 6. In order to describe a more general case, Equation (9) is expanded by two rotation matrices resulting in Equation (10) [15]:

(10) $${\vec{f}}_{rx}=\left(\frac{C}{\varrho^{3}}\right){T_{\alpha }}^{-1}{T_{\beta }}^{-1}ST_{\beta }T_{\alpha }{\vec{f}}_{tx},$$

where $T_{\alpha }$ describes the rotation matrix for rotations around the *z*-axis and $T_{\beta }$ the rotation matrix for rotations around the *y*-axis. Figure 7 shows this more general arrangement.

**Figure 8:**
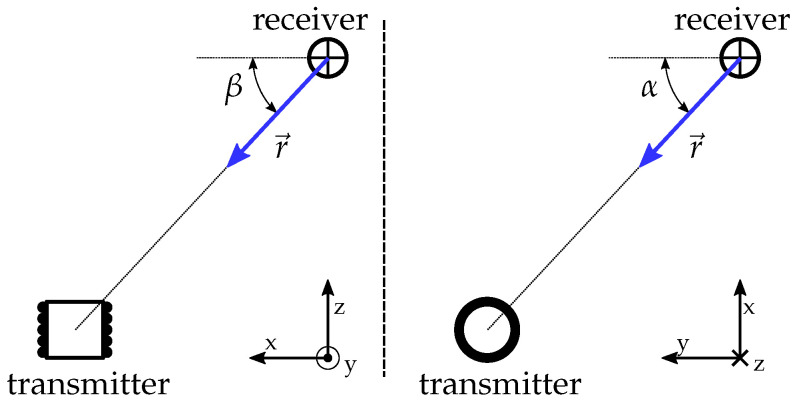
General transmitter–receiver arrangement and the directional vector $\vec{r}$ [21].

A directional vector $\vec{r}$ which points from the receiver to the position of the transmitter can be calculated by measuring the three *x*-, *y*-, and *z*-components of the magnetic field at a defined position. The measured values are the components of the vector $\vec{f_{rx}}$. Equation (10) is then solved for the angles α and β. The direction vector $\vec{r}$ is fully defined by those two angles using the spherical coordinate system (see, therefore, Figure 8).

Our transmitter consists of one single coil with an unknown transmission power. The transmission power will depend on the transmitter position in the reactor and is assumed to be unknown since its power supply can fluctuate due to the not perfectly homogeneous supply magnetic field. Because of that, in order to solve the coupling Equation (10), we have to simplify our model. With the assumption that the transmitter coil is always aligned with a global coordinate, in our case, the global *z*-coordinate (the transmitter signal vector $\vec{f_{tx}}={\left(0\;0\;a\right)}^{T}$ is consequentially an unknown value *a* in the *z*-direction), Equation (10) can now be solved since we have three equations and three unknown variables: α, β, and *a*. If our transmitting coil would not be aligned with the global *z*-coordinate, the coupling equation has to be expanded with additional rotary matrices in order to include the orientation of the transmitter. The additional unknown angles would prevent the coupling equation from being analytically solvable.

Due to the unknown transmitting power, the constant factor *C* and the factor $\varrho^{3}$ can be omitted. This does not affect the solvability of the coupling equation since we do only calculate a directional vector and not a vector with a defined length. Two or more receivers are needed in order to locate the transmitter. *MATLAB* is used to solve the inverse problem. In doing so, the magnetic field values of the transmitter coil measured with a receiver at a defined position are passed to Equation (10); the angles α and β and the value *a* are passed to it as unknown variables for which the equation is then solved. This is done for at least one additional receiver in order to get a second directional vector. Those vectors need to be transformed from the coordinate system of the respective receiver to a global coordinate system of the used measurement setup. Once the directional vectors are defined in the same coordinate system, the position of the transmitter is calculated by finding the point where the two directional vectors come closest, in an ideal case their intersection [21].

## 4. Measurement Hardware

In order to perform localization measurements for proving the feasibility of the described tracking methodology, a transmitter and at least two receivers are needed. In this section, the transmitter design as well as the receiver design and the measurement setup are described.

### 4.1. Design of the Transmitter

**Figure 9:**
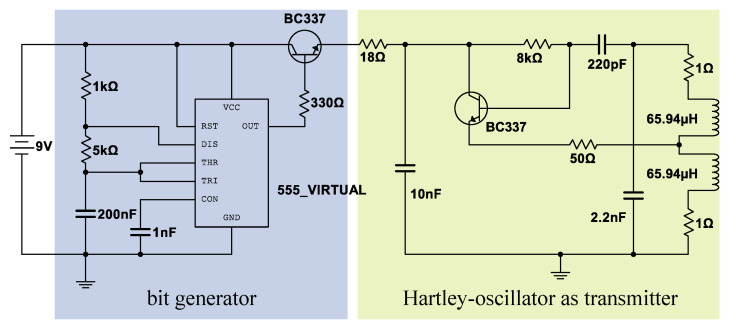
Transmitter circuit: bit generator based on the integrated circuit LMC555 and the Hartley-oscillator [18].

For this first step, we designed a transmitter without a real sensor. Instead of a sensor, we use a square wave generator realized with the timer integrated circuit LMC555 to simulate a digital sensor data stream. The high level of the square wave signal represents the 1-bit and the low level the 0-bit. As used by Edelmann et al. [17], our main transmitter architecture is also a Hartley-oscillator where its inductance is used as transmitter coil. In this first step, the on-off keying is applied to modulate the simulated data stream on the carrier signal. Figure 9 shows the circuit of this first prototype. The transmitting coil is a round coil with 115 turns wounded on a 3D-printed socket. The current in the coil is approx. 50mA with an inductance of 132H. The transmitting frequency is set to 295kHz. The Hartley-oscillator is switched on and off using the square wave signal generated by our bit generator [18]. In this first step, the transmitter is supplied with energy via a cable.

### 4.2. Receiver Architecture

Like that described in the previous Section 3, in order to calculate a directional vector for each receiver by solving Equation (10), receivers that are able to measure the transmitter magnetic field in all three spatial directions at a defined position are needed.

#### 4.2.1. Main Receiver Design

**Figure 10:**
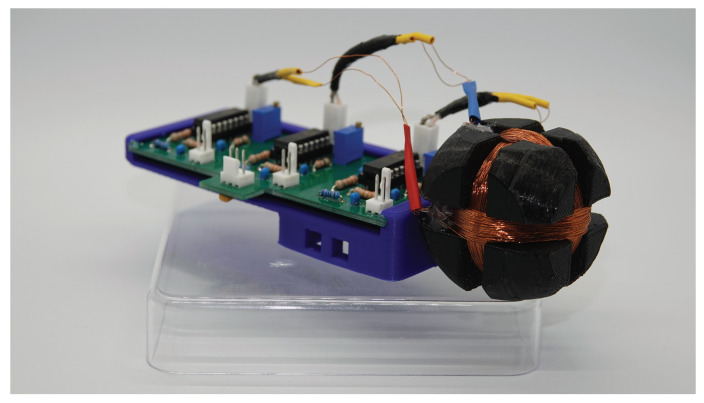
Receiver with the orthogonal aligned coils.

The main requirement to our receivers is the ability to measure the magnetic field in the three *x*-, *y*-, and *z*-directions. To meet these requirements, three receiver coils per receiver are needed. By placing these three coils with their axis orthogonal to one another and using the same coil center point for each coil, this requirement can be met. Figure 10 shows the coil alignment of our receiver. A second benefit of that architecture are the negligibly low mutual inductions of the three coils, in theory zero. This fact can be seen in Equations (6) and (7). At an angle of $\zeta =90^{\circ }$, the radial part of the magnetic field becomes zero and the tangential part of the field points in the direction of the coil axis. Due to the orthogonality of the coil axes and according to the inductance law, the tangential part at the given angle can not induct a current in the other two coils.

#### 4.2.2. Receiver Electronics

**Figure 11:**
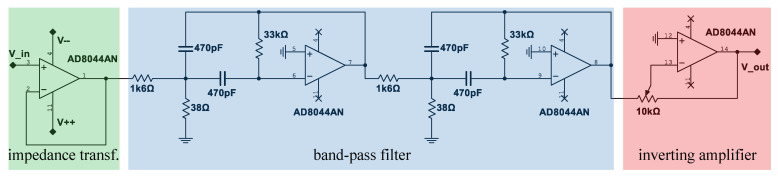
Filter- and Amplifier-circuit used for each receiver coil.

**Figure 12:**
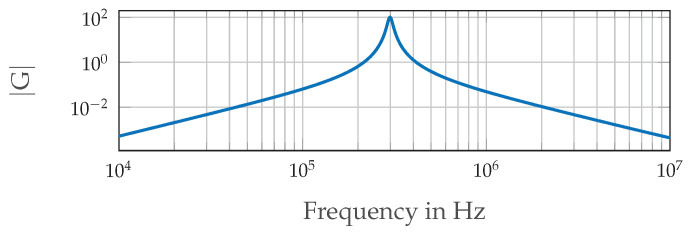
Frequency response of the filter circuit shown in Figure 11.

The coil signals are amplified and filtered before being digitized by the I-O device. Therefore, we use an inverting amplifier and two second order band-pass filters with multiple negative feedback connected in series. Together, this results in a fourth order filter. The filters are tuned to the transmitter frequency. Figure 11 shows the used electric circuit, and the Bode-plot in Figure 12 shows its amplitude response |G| where the gain factor of the inverting amplifier is set at 1. The inverting amplifier is needed in order to match the output amplitudes of the three circuits (one for every coil) to each other. The single filters are dimensioned according to (Tietze et al. [22] p. 820–822). The component values shown in Figure 11 were calculated for a Q-factor of 15 and the gain factor $|A_{r}|=10$ for each single band-pass. This results in a total gain factor of $|A_{r}|=100$ at the resonant frequency, which can be seen in Figure 12. The achievable Q-factor of a single filter is limited by the real properties of the operational amplifier: its open-loop gain needs to be large compared to $2Q^{2}$ [22].

As can be seen in the circuit diagram in Figure 11, each receiver coil is followed by an impedance transformer and is therefore connected directly to the input of the operational amplifier. Since the current at the input of an operational amplifier is nearly zero, no current flows through the receiver coil. The magnetic flux of a coil results in ϕ=μ·n·A·I, and it can be seen that it turns to zero if the current *I* turns to zero and so does the mutual influence between the three receiver coils.

### 4.3. Measurements Setup

**Figure 13:**
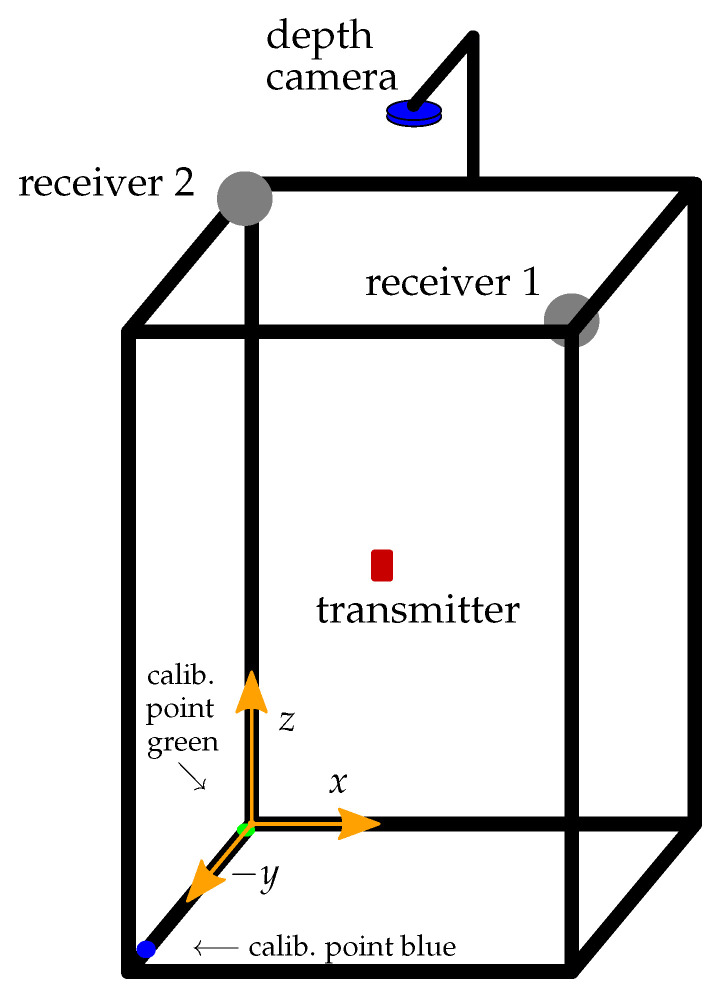
Measurement setup used to perform the location measurements.

The setup shown in Figure 13 is used to perform the localization measurements referred to later in this paper, in Section 6. The setup is completely made of plastic materials in order to prevent the setup construction to influence the magnetic field of the transmitter. Two receivers are used to measure the transmitter magnetic field and thus to locate its coil. We use the *NI USB-6366* (National Instruments, Austin, TX, USA) I/O device for digitalizing the receiver signals and *MATLAB* for solving the coupling equation Equation (10). The setup shown in Figure 13 and thus also the region of interest for the measurements is 50cm wide (x) and high (z) and 30cm deep (y). Due to the rotation symmetry of the magnetic dipole field and since we do not track the phase of the receiver signals for now, we get multiple solutions for the angles α and β (which are used to define the directional vector $\vec{r}$ pointing from a receiver to the transmitter) by solving Equation (10). To counteract this problem, the receivers are each positioned in a corner of the region of interest; in doing so, only one solution for the directional vector will point into the room of interest.

## 5. Calibration of the Receivers by Means of a LIDAR Depth Camera

### 5.1. Calibration Methodology

In order to calibrate our position measurement system, the *Intel Realsense Lidar Camera L515* (Intel, Santa Clara, CA, USA) depth camera is used. The transmitter position measured with the camera is assumed to be the exact one and is used as a reference to calibrate the system. Therefore, by knowing the exact receiver positions, the receiver signal vector is calculated for each one using the transmitter position measured with the camera. The calculated signal vector is then compared with the signal vector measured with the receiver. Figure 13 shows the measurement setup with the depth camera at the top pointing into the region of interest. The used camera has an RGB module in addition to the LIDAR module. This facilitates the determination of the coil position. Therefore, a red marker is placed on the transmitting coil and the ratio between the three color channels is considered in order to find the position of the coil in the RGB image. Using the determined pixel coordinates, the measured coil position can be taken from the point cloud registered by the LIDAR module. According to the data-sheet of the *Intel Realsense Lidar Camera L515* camera, the depth accuracy at a distance lower than 1m is below 5mm when using the coarse VGA resolution (640 per 480 pixel). In our setup, the distances between the camera and the transmitter are always lower than 1m; additionally, the resolution of the depth camera is set to XGA (1024 per 768 pixel), which further increases the accuracy.

The calibration procedure is done in two steps. In the first step, the amplitude ratio between the three receiver coils is adjusted for each receiver. This can be done by adjusting the gain factor of the inverting amplifier in the receiver circuit shown in Figure 11. The transmitter is therefore placed in our region of interest and, by comparing the measured and calculated amplitude ratios, the gain factor of each receiver channel is adjusted. The second step in order to compensate inaccuracies of the receivers and influences from the environment like e.g., near conducting materials, is to create a so-called calibration map. In order to generate this map, a grid of transmitter positions is defined in the region of interest. For each of them, the position is measured by using our inductive localization method as well as the depth camera. For all grid points, a displacement vector is calculated by subtracting the position coordinates measured with the camera from the coordinates measured with the inductive method. These vectors are saved in our calibration map at each inductively measured position. For further measurements, the inductively measured position is improved by searching the three nearest points in the calibration map and by calculating a weighted mean value for each coordinate deviation.

### 5.2. Automatic Calibration of the Camera

In order to convert the positions measured with the depth camera from the coordinate system of the camera to the coordinate system of our measurement system, the camera position as well as the camera orientation need to be defined. The used *Intel Realsense Lidar Camera L515* depth camera has an inertial measurement unit that is used to detect the azimuth and tilt angle of its orientation. The third orientation angle is calculated using two calibration points on the measurement setup frame (see Figure 13). The first calibration point has the color green and the second the color blue, and the points are detected using the RGB module of the depth camera. The ratio of the three color channels of the RGB image is used to identify the two calibration points. In knowing the exact position of the calibration points, the third orientation angle and the position of the camera can be calculated in order to define the exact transformation matrices to transform the measured transmitter positions from the camera coordinate system to the coordinate system of the measurement setup.

### 5.3. Improvement of the Inductively Measured Positions by Means of the Calibration Map

The inductively measured position is improved by searching for the three nearest calibration points in the calibration map. The distance between the inductively measured position and the single calibration points is calculated. The reciprocal of those calculated distances is saved as weights in a data array $w_{i}$ that is reshaped in order to adopt values between 0 and 1; in doing so, the vector needs a fourth entry with the value 0; otherwise, the reciprocal of the bigger distance value would always be reshaped to 0. The improved position is calculated with the following equation:

(11) $${\vec{P}}_{calib}={\vec{P}}_{ind}+\left(\begin{array}{c} \frac{1}{\sum_{i=1}^{3}w_{i}}\left(w_{1}d_{1x}+w_{2}d_{2x}+w_{3}d_{3x}\right)\\ \frac{1}{\sum_{i=1}^{3}w_{i}}\left(w_{1}d_{1y}+w_{2}d_{2y}+w_{3}d_{3y}\right)\\ \frac{1}{\sum_{i=1}^{3}w_{i}}\left(w_{1}d_{1z}+w_{2}d_{2z}+w_{3}d_{3z}\right) \end{array}\right),$$

where ${\vec{P}}_{calib}$ is the improved position, ${\vec{P}}_{ind}$ the inductively measured position, $w_{i}$ the weights, and ${\vec{d}}_{1-3}$ the three nearest difference vectors.

## 6. Results

### 6.1. Receiver Signals

**Figure 14:**
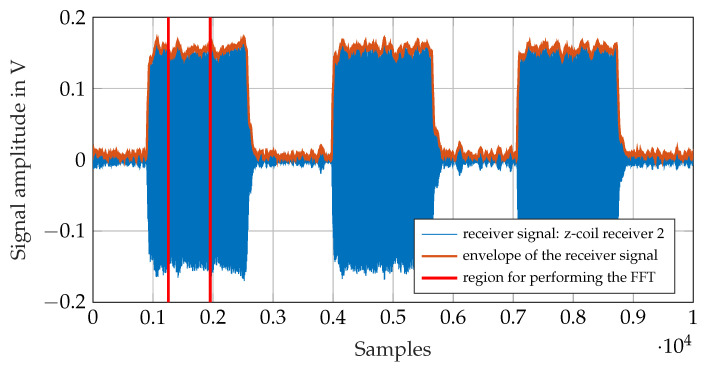
Receiver signal: signal at the z-coil of the receiver 2.

**Figure 15:**
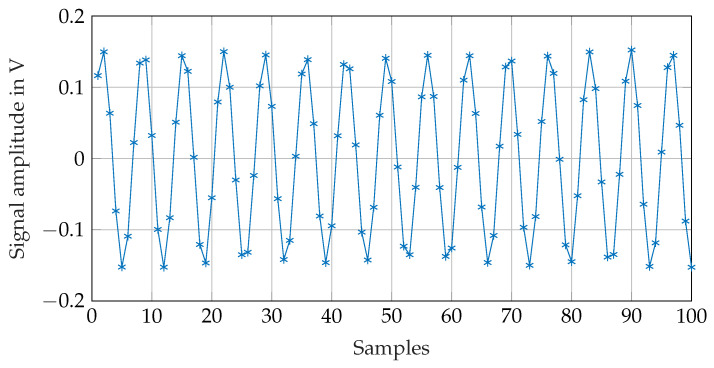
Receiver signal: first 100 samples of the marked region in Figure 14.

Figure 14 shows a sample of the recorded receiver signal. It is the received signal at the z-coil of receiver 2 (see Figure 13). Figure 15 shows a detail of the digitalized signal. With the I/O device sample rate of 2MS/s, the received signal at a frequency of 295kHz can be digitalized with ≈6.8 samples pro per period. The amplitude of the signal is calculated using the Fast Fourier Transformation (FFT). The FFT is not performed on the whole signal showed in Figure 14 but just on 700 samples during the high period of the on-off coded signal. The envelope of the received signal is therefore calculated in order to find the high period of the on-off-keyed signal. In addition, 700 samples of the received signal are selected from the high level of the on-off coded receiver signal as marked in (Figure 14), and the FFT is calculated from this.

**Figure 16:**
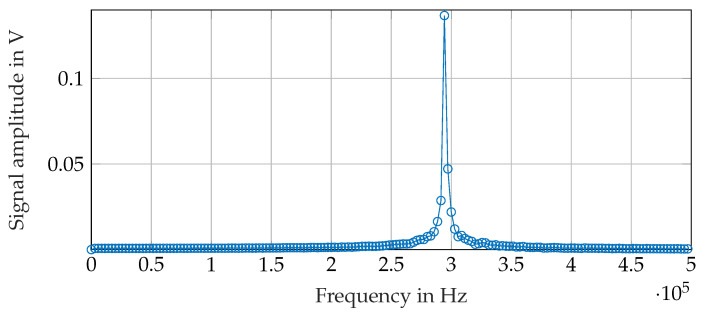
Receiver signal: Fast Fourier Transformation of the marked region in Figure 14.

Figure 16 shows the FFT of the signal shown in Figure 14. The signal amplitude at the transmitter frequency is calculated by searching the peak at the desired frequency in the FFT. The transmitter position is calculated with the equations from Section 3 using the so calculated receiver signal amplitudes.

### 6.2. Calibration Map

**Figure 17:**
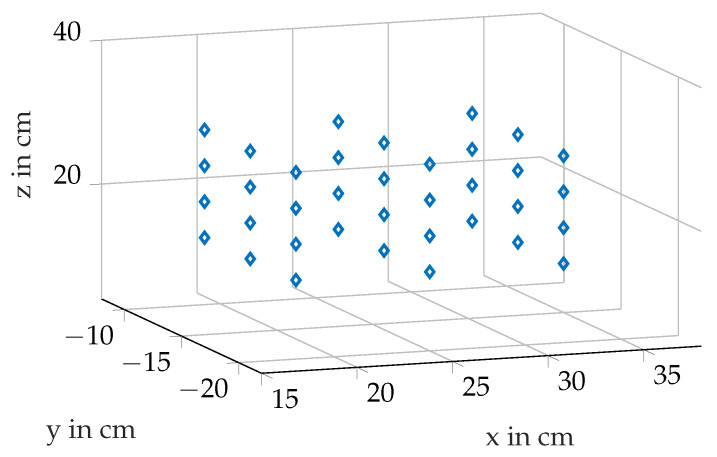
36 chosen points for the demo calibration map.

In order to represent the calibration map more easily, it was generated for a smaller area inside our room of interest. The chosen points for the calibration map for the small region inside the measurement setup are shown in Figure 17.

In performing the measurements for generating the calibration map, the transmitter is positioned manually to the desired positions. In order to calculate an accurate difference vector between the real position and the inductively measured one, the exact position of the transmitter is measured with the depth camera so the inaccuracies due to the manual way of positioning the transmitter are bypassed.

**Figure 18:**
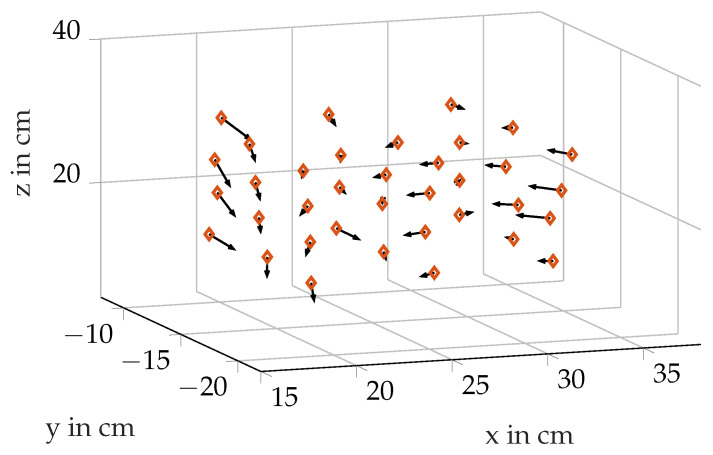
Calibration map points and the displacement arrows.

The difference vectors are shown in Figure 18. The displayed vectors are normalized to the length 1 in order to enable a better representation. The shown calibration map is used to tune the inductively measured positions.

### 6.3. Localization Results

Table 2 shows the achieved results for five randomly chosen positions. The difference between the position coordinates measured with the depth camera and the inductively measured positions are given as ${\vec{\Delta }}_{cam-ind}$, and, consequentially, ${\vec{\Delta }}_{cam-calib}$ indicates the differences between the tuned position and the exact position coordinates.

## 7. Discussion

**Figure 19:**
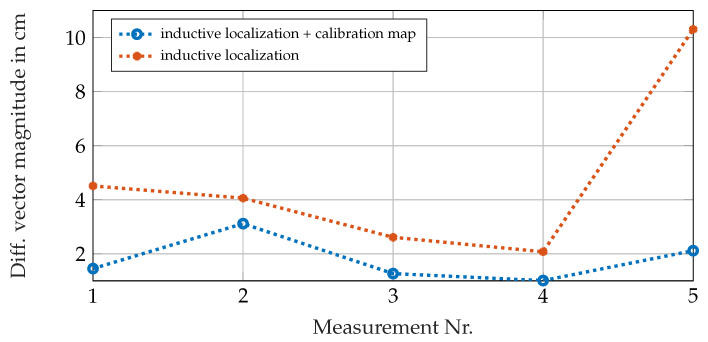
Lengths of the difference vectors between the measured position and the real position for just the inductive measurement system and the improvement of the results in using the calibration map.

The functionality of the methods described in this paper could be demonstrated in Section 6. According to Table 2, the distances between the exact position coordinates and the inductively measured ones are reduced in using the calibration map. Figure 19 compares the magnitude of the difference vectors ${\vec{\Delta }}_{cam-ind}$ and ${\vec{\Delta }}_{cam-calib}$ for each five positions given in Table 2. It can be seen that the inaccuracies decrease significantly in using the calibration map.

The accuracy of the inductive position measurement system presented here can be improved in using more than two receivers. We already performed preliminary simulations where a noticeable increase in the localization accuracy could be seen by adding one more receiver [23]. The chosen positions of the receivers may also have an impact on the overall accuracy of the inductive localization method. Optimization studies in minimizing the difference vectors between the right position and the recalculated positions in simulating noisy receiver signals will be done in order to get an insight into whether there are certain positions or position combinations of the receivers for which the general localization accuracy can be increased. The localization task at the moment is computed with the constraint that the transmitting coil is aligned with the (vertical) *z*-axis. The alignment with the *z*-axis is also a needed constraint for an optimal energy transmission. For the wireless illumination, this problem was solved in setting the center of gravity of the WLE in a low position inside of it. In doing so, the WLEs align themselves while floating around in the photoreactor [5]. The same approach can be used for the wireless sensors. An additional approach to calculate the position of the wireless sensors, also if they are not perfectly aligned with the *z*-axis, could be to find the minimum in the coupling equation between the receiver and the transmitter expanded by rotation matrices in order to consider the orientation angles. The position calculated with the simplification of a vertical transmitter coil can therefore be used as the initial value. There is no need for searching the minimum of an expanded coupling equation if the transmitter transmits its signal in more than one spatial direction using different frequencies or different times for each direction since we then would have an equation system with the same amount of known and unknown variables also for a misaligned transmitter. In [24], a methodology is shown where a biaxial sensor is tracked with a triaxial transmitter.

Regarding the wireless communication, for now, we just transmit the simulated sensor bits in using a square wave signal to switch the Hartley-oscillator on and off. The research focus in this step was on the localization task. The next major step will be the validation of the used modulation technique in order to transmit real measured sensor data. In a similar project, in order to send the measured data, the 434MHz frequency band is used. The wireless sensor spheres for monitoring biological processes presented by Lauterbach et al. [25] are powered through an accumulator placed in the center of the sensor sphere. The sensor spheres can not be located and the measurement frequency needs to be adapted to the process runtime because of the limited capacity of the accumulator [25]. Our conception will provide for an energy supply of the sensors through the inductive power supply used for the WLEs. Considering Equation (2) in Section 2, a drawback in using higher frequencies like e.g., the 434MHz frequency band can be seen. The transmission range decreases with higher frequency and, in addition, due to the shorter wavelength, the quasi static field approach used for the localization task would no longer be valid.

In order to use the inductive energy transmission of the internal illumination also for the wireless sensors, it is the idea to utilize the transmitter coil for the data transmission as well as for supplying the transmitter with energy. Therefore, the coil needs to be switched between two circuits: the transmitter oscillator and a second oscillator tuned to the 178kHz of the wireless power supply. This second LC-tank will be used to charge energy storage like, e.g., a capacitance which will supply the transmitter with energy during the phase in which the transmitter transmits the measured data.

The magnetic field generated by the driving coils of the power supply will be an obstacle for the transmitter while sending the measured data. Even if the oscillator circuit is tuned to the transmitting frequency of 295kHz, the supply field will prevent the oscillator from oscillating at the desired frequency. Preliminary spice-simulations confirmed this fact. To overcome this issue, a filter circuit connected to the LC-tank of the transmitter oscillator based on passive electrical components is needed in order to conduct the voltage inducted at 178kHz to the ground. The filter must have a low impedance at the named frequency and a high impedance at 295kHz. At this frequency, the phase of the filter must be zero in order to maintain the phase condition of the oscillator circuit. This characteristic can be achieved in switching a capacitance in parallel with a series connection of an inductance and a capacitance.

The potential application of the described inductive tracking methodology is not limited to internally illuminated photoreactors. It is suitable for application areas in which locating with conventional optical methods or methods which use higher-frequency electromagnetic signals are not suitable. A restriction is given by the eventual presence of materials in the vicinity of the system, which could impair the propagation characteristics of the magnetic field of the transmitter coil and thus have to be taken in account. The localization task in using quasi static fields gains in importance also for larger scale environments like e.g., the indoor navigation [26], which can, e.g., be used for emergency responders in order to locate them [27].

## 8. Conclusions

In this paper, a methodology for localizing wireless sensors in photobioreactors is discussed. Due to the promising propagation properties of magnetic fields in underwater environments, an MI system is used for the communication and for the localization task. The used transmitter and receiver architecture is shown as well as the used measurement setup. The analytical model used to describe the magnetic field of the transmitter coil is compared with simulation results in order to validate it. A calibration method for the system in using a LIDAR depth camera is presented. Test location measurements were performed in order to demonstrate the feasibility of the described methodology. The accuracy of the inductive localization is additionally improved in using a calibration map. A localization accuracy up to approx. 3cm was shown.

## Figures and Tables

**Table 1:** Calculated NRMSE values.

	Radial Distance 25 mm	Radial Distance 50 mm	Radial Distance 250 mm
NRMSE	0.2541	0.0267	0.0258

**Table 2:** Real positions, measured transmitter-positions, and tuned transmitter-positions.

Nr.	Coordinates	${\vec{P}}_{\mathrm{cam}}$	${\vec{P}}_{\mathrm{ind}}$	${\vec{\Delta }}_{\mathrm{cam}-\mathrm{ind}}$	${\vec{P}}_{\mathrm{calib}}$	${\vec{\Delta }}_{\mathrm{cam}-\mathrm{calib}}$
	X	22.3cm	23.0cm	−0.7cm	22.1cm	0.2cm
1	Y	−14.1cm	−13.4cm	−0.7cm	−14.4cm	0.3cm
	Z	26.9cm	22.5cm	4.4cm	25.5cm	1.4cm
	X	29.0cm	26.9cm	2.1cm	26.4cm	2.6cm
2	Y	−18.4cm	−15.2cm	−3.2cm	−16.8cm	−1.6cm
	Z	21.8cm	20.4cm	1.4cm	22.5cm	−0.7cm
	X	29.1cm	30.2cm	−1.1cm	28.2cm	0.9cm
3	Y	−13.9cm	−12.2cm	−1.7cm	−13.1cm	−0.8cm
	Z	15.2cm	13.5cm	1.7cm	14.8cm	0.4cm
	X	31.0cm	31.5cm	−0.5cm	30.7cm	0.3cm
4	Y	−11.9cm	−11.4cm	−0.5cm	−11.6cm	−0.3cm
	Z	29.3cm	27.4cm	1.9cm	28.3cm	1.0cm
	X	15.4cm	17.0cm	−1.6cm	15.8cm	−0.4cm
5	Y	−15.7cm	−15.3cm	−0.4cm	−15.9cm	0.2cm
	Z	29.3cm	19.1cm	10.2cm	27.2cm	2.1cm

## Abbreviations

The following abbreviations are used in this manuscript:

NRMSE	Normalized Root-Mean-Square Error
FFT	Fast Fourier Transformation
LIDAR	Light Detection and Ranging
MI	Magnetic Induction
WLE	Wireless Light Emitter
